# Supplementary material for: The Transcription Factors TaTDRL and TaMYB103 Synergistically Activate the Expression of TAA1a in Wheat, Which Positively Regulates the Development of Microspore in Arabidopsis
Source: Int J Mol Sci. 2022 Jul 20;23(14):7996. doi: 10.3390/ijms23147996 (PMC9321142; doi:10.3390/ijms23147996)
Supplement: Supplementary file 1 [file ijms-23-07996-s001.zip › Supplementary Tables.pdf]

Table S1. Primers used for quantitative real-time RT-PCR analysis

Primer	Sequence (5′-3′)
TAA1a-qPCR-F	GCCACCACCAACTTCTACGA
TAA1a-qPCR-R	CACCTTGAGATTGGGGCACT
TaTDRL-qPCR-F	CAACGACCGCCTCTACAAG
TaTDRL-qPCR-R	GCTCCTTCACCTGCTTCTG
TaMYB103-qPCR-F	GAGTTCACTGACGCCGAG
TaMYB103-qPCR-R	AGAAGGACTTGTGCGTGAC
TaActin-qPCR-F	ACCTTCAGTTGCCCAGCAAT
TaActin-qPCR-R	CAGAGTCGAGCACAATACCAGTTG
MS2-qPCR-F	CATGCATGGACTCTAAAG
MS2-qPCR-R	CATCATCTTACTATCCAC
EF1α-qPCR-F	TCGCGTGTCTGTGTTCTTGT
EF1α-qPCR-R	GTGTGTGTAGATCCGCCACC

Table S2. Primers used for genes cloning in this study

Primer	Sequence (5′-3′)
TaTDRL-F	TGGGAGGAGGAGATTATCACC
TaTDRL-R	ATCCATGGCGAGGTACTGCAG
TaMYB103-F	ATGGGCCGGATCCCGTGCTGCG
TaMYB103-R	GTCACACATGTGATTCGTCAGC

Table S3. Primers for gene-specific and plasmid construction in this study

**Primer**	**Sequence (5′-3′)**
	**Primers for subcellular localization plasmids**
TaTDRL-EGFP-F	CGAGCTCAAGCTTCGAAATGGGAGGAGGAGATTATCACC
TaTDRL-EGFP-R	CGACTGCAGAATTCGAAATCCATGGCGAGGTACTGCAG
TaMYB103-EGFP-F	CGAGCTCAAGCTTCGAAATGGGCCGGATCCCGTGCTGCG
TaMYB103-EGFP-R	CGACTGCAGAATTCGAAGTCACACATGTGATTCGTCAGC
	**BiFC plasmids primers**
TaTDRL-nEYFP-F	CGAGCTCAAGCTTCGAAATGGGAGGAGGAGATTATCACC
TaTDRL-nEYFP-R	CGACTGCAGAATTCGAAATCCATGGCGAGGTACTGCAG
TaMYB103-cEYFP-F	CGAGCTCAAGCTTCGAAATGGGCCGGATCCCGTGCTGCG
TaMYB103-cEYFP-R	CGACTGCAGAATTCGAAGTCACACATGTGATTCGTCAGC
	**Primers for plant expression plasmids**
TaTDRL-OE-F	GGGGACTCTTGACCATGGAGATGGGAGGAGGAGATTAT
TaTDRL-OE-R	TTCTTCTCCTTTACTAGTTTACTTATCGTCGTCATCCTTGTAATCATCCATGGCGAGGTA
TaMYB103-OE-F	GGGGACTCTTGACCATGGAGATGGGCCGGATCCCGTGC
TaMYB103-OE-R	TTCTTCTCCTTTACTAGTTTACTTATCGTCGTCATCCTTGTAATCGTCACACATGTGATT
TaTDRL-EAR-F	GGGGACTCTTGACCATGGAGATGGGAGGAGGAGATTAT
TaTDRL-EAR-R	TTCTTCTCCTTTACTAGTTTAAGCGAAACCCAAACGGAGTTCTAGATCCAGATCCAGATCCATGGCGAGGTA
TaMYB103-EAR-F	GGGGACTCTTGACCATGGAGATGGGCCGGATCCCGTGC
TaMYB103-EAR-R	TTCTTCTCCTTTACTAGTTTAAGCGAAACCCAAACGGAGTTCTAGATCCAGATCCAGGTCACACATGTGATT
	**Primers for Dual-luciferase assay**
proTAA1a::LUC-F	GTCGACGGTATCGATAAGCTTCTAGTCTCTTACTACTGAGTCC
proTAA1a::LUC-R	CGCTCTAGAACTAGTGGATCCAACCATCTTGATTCTTTTCT
TaTDRL-62-SK-F	CGCTCTAGAACTAGTGGATCCATGGGAGGAGGAGATTATCACC
TaTDRL-62-SK-R	GTCGACGGTATCGATAAGCTTATCCATGGCGAGGTACTGCAG
TaMYB103-62-SK-F	CGCTCTAGAACTAGTGGATCCATGGGCCGGATCCCGTGCTGCG
TaMYB103-62-SK-R	GTCGACGGTATCGATAAGCTTATCCATGGCGAGGTACTGCAG
	**Primers for yeast one-hybrid assay and yeast two-hybrid assay**
TaTDRL-BD-F	GCCATGGAGGCCGAATTCATGGGAGGAGGAGATTATCACC
TaTDRL-BD-R	CGGCCGCTGCAGGTCGACTCAATCCATGGCGAGGTACTGC
TaTDRL-NBD-F	GCCATGGAGGCCGAATTCATGGGAGGAGGAGATTATCACC
TaTDRL-NBD-F	CGGCCGCTGCAGGTCGACGTTCTTGCACTGCTGCCGCTTC
TaTDRL-HLHBD-F	GCCATGGAGGCCGAATTCCTCGAGGCGGAGCGGAAGCGGC
TaTDRL-HLHBD-R	CGGCCGCTGCAGGTCGACCTCCTTCACCTGCTTCTGCAGC
TaTDRL-CBD-F	GCCATGGAGGCCGAATTCCTGCAGGACGAGCTGGAGGACC
TaTDRL-CBD-R	CGGCCGCTGCAGGTCGACTCAATCCAT GGCGAGGTACTGC
TaTDRL-NHLHBD-F	GCCATGGAGGCCGAATTCATGGGAGGAGGAGATTATCACC
TaTDRL-NHLHBD-R	CGGCCGCTGCAGGTCGACCTCCTTCACCTGCTTCTGCAGC

TaTDRL-HLHCBD-F	GCCATGGAGGCCGAATTCCTCGAGGCGGAGCGGAAGCGGC
TaTDRL-HLHCBD-R	CGGCCGCTGCAGGTCGACTCAATCCATGGCGAGGTACTGC
TaMYB103-NBD-F	GCCATGGAGGCCGAATTCATGGGCCGGATCCCGTGCTGCG
TaMYB103-NBD-R	CGGCCGCTGCAGGTCGACGAACTCCGTCGCCTGCGCGGGG
TaTDRL-AD-F	GGGGACTCTTGACCATGGAGATGGGAGGAGGAGATTAT
TaTDRL-AD-R	AGCTCGAGCTCGATGGATCCTCAATCCATGGCGAGGTACTGC
TaMYB103-AD-F	CCATGGAGGCCAGTGAATTCATGGGCCGGATCCCGTGCTGC
TaMYB103-AD-R	AGCTCGAGCTCGATGGATCCTTAGTCACACATGTGATTCGT
TaTDRL-pAbAi-F	AAGCTTGAATTCGAGCTCAAACACATGGCAAAACACATGGCAAAAC ACATGGCAGTCGACCTCGAGGCATGT
TaTDRL-pAbAi-R	ACATGCCTCGAGGTCGACTGCCATGTGTTTTGCCATGTGTTTTGCCATG TGTTTGAGCTCGAATTCAAGCTT
TaMYB103-pAbAi-F	AAGCTTGAATTCGAGCTCAGTCCAACCTTAAGTCCAACCTTAAGTCCA ACCTTAGTCGACCTCGAGGCATGT
TaMYB103-pAbAi-R	ACATGCCTCGAGGTCGACTAAGGTTGGACTTAAGGTTGGACTTAAGG TTGGACTGAGCTCGAATTCAAGCTT

Note: Underline as enzyme cutting site
