# Supplementary material for: The Transcription Factors TaTDRL and TaMYB103 Synergistically Activate the Expression of TAA1a in Wheat, Which Positively Regulates the Development of Microspore in Arabidopsis
Source: Int J Mol Sci. 2022 Jul 20;23(14):7996. doi: 10.3390/ijms23147996 (PMC9321142; doi:10.3390/ijms23147996)
Supplement: Supplementary file 1 [file ijms-23-07996-s001.zip › Supplementary Figures.pdf]

-1309 CTAGTCTCTTACTACTGAGTCCCCTATCATTTTCCCATATGTAAAACGACGACACGTGGTCGGCCGGTGGGCATCTAAT
TCT-motif (-)GATA-motif E-box TGA-element E-box

-1230 GCCTTAATGCATCTCCCAACCGATGATCCGTTGGGGAGGTTTCCTTCATGTTTACTAAACAAAAGGAGTAGACCTTGGTA
MYB MYB103 specific-recognizes motif MYB recognition site

-1150 GATGATGCGCCGCCTCTCTTGATGGTTCTTGTTCTGTTGTCGTCTTCCTATCTCATCCCATCAATGTTAGCGTTGTGCAA
GCC-BOX GARE-motif CAAT-box

-1070 GCAAGTGGTTAAGTGGAAGGAACCATGCGTTTTGAACTTCTGATAAACCATGGAGTAGATGGAATTAGTAGGCGCCACAC
E-box I-BOX SORLIP

-990 ATAGCAGATCTATGCTGCCCGTGCGTACCGGGAATTACTAGTGCAGGCCTCTTTAAGTCGGTCAGGTACGTAGCACTCAA
ABRE

-910 CCAAACAAGCAGGACATGTTTGAAAATCACTATTCTAACTTTAATAATGTACACAAGGAATTGAAGGCATGATAAATTCT
MYB

-830 ATGCTGGTACCACCCATGCGACTGGCGCTCGTTATGAAGTACTAGTAGAGTAGAGTACTAACATGATGATCTTCAATATG
CAAT-box

-750 TTGTGTCCAAGTTCTTTGCCACAGGAGCAAAAATATACCACTGAATTATATGCTAATAGTGTAATTATTGGCCATTTTTT
SORLIP TATA-box TATA-box (-)BOX4

-670 GTTCTAATGAGAGAAAAATACGCTATGCGTTTCCAATGGATGGAGTACGATACTACTCTATCATACTCCACTCATCAATT
MYB POLLEN1LELAT52 CAAT-box E-box

-590 GTAGGAGTACCCTACAATTTCTAATGGTATAACTATGATGACATGCACTAATATTTGTCAAGTAAATCTTTGGTGAAAA
CAAT-box MYB TATA-box RY repeat GTGA-motif

-510 TTTAGCACAAATTACAATGAAGACTAATAAACCAGGACGGAGGTAGTAGCAATAATACTGAATACTGCTGGTTGTCCACC
CAAT-box MYB CAAT-box E-box

-430 TGAACACAATTAATATGATGGCTAGCAACGTACCGCAAACCCAATAATAACCCTGTTTGAAAATCTAGAGTACTTCTACG
CAAT-box ACE CAAT-box G-box

-350 CGTTATTTTGACCGCATGTGATTCCTTTGATGATCACTGGGTCCTTGTTTTGGTTAGGCAGCGCATAAATGCTGCGCTCT
W-box E-box

-270 TAAGTGCCTCAAATTAAGACTCCCCTCTCCTACCTACAACAAAACACCTTTTTTGAGCAAAAGATGGGCCACCCCGTCCG
SORLIPS A-box

-190 ATTTCATATAATGAAACTAACTGGTTCAACTGCCAAACAACAAACCACAAGACAAAACACCTTGATGGCCCGTGTATAAA
TATA-box E-box MYB TATA-box

-110 TATTGTCTACATGACCCTCTTTCCACTCACCTA[CACACAAATCACCCCCCCCCCCCCCCCCCTCCTCTCTCTCTCTCT
W-box

-30 CTCTCGCACGCACA]CAAGAAAAGAATCAAG<u>ATG</u>
POLLEN1LELAT52 +1

Figure S1. Analysis of the *TAA1a* promoter sequence from the wheat fertile near-isogenic line MF(KOTS-90-110). The translation initiation site "ATG" (marked in underline) of *TAA1a* was numbered as +1. The CAAT and TATA boxes were highlighted in bright green and yellow, respectively. The plant hormone response elements were highlighted in red, pollen-specific, light-responsive, stress response, E-box and MYB103 specific-recognizes elements highlighted in lilac, orange, brown, light blue and purple , respectively. The Unpublished sequences of *proTAA1a* in the Ensembl Plants online database was indicated by "[ ]".

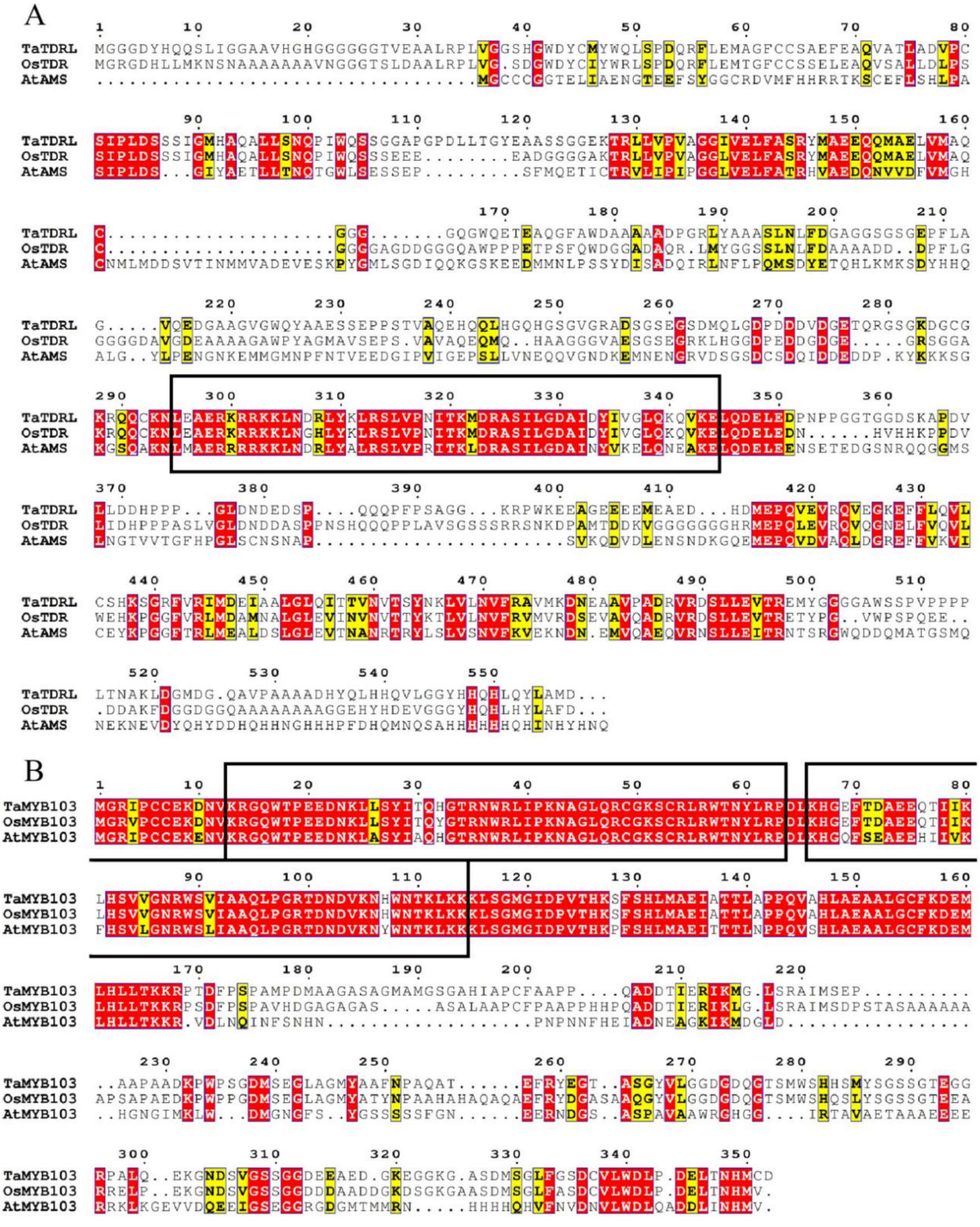

Figure S2. Protein sequence analysis of TaTDRL and TaMYB103 protein ; (A) Comparison of amino acid sequences of TaTDRL with their close homologs. The accession numbers for the aligned protein are: *OsTDR* (Os02g0120500), *AtAMS* (AT2G20180.1); the black frame shows the bHLH domain (295-344 aa); (B) Comparison of amino acid sequences of TaMYB103 with their close homologs. The accession numbers for the aligned protein are: *OsMYB103* (XP_015635420.1), *AtMYB103* (NP_200422.1); two black borders show the SANT domain (13-63 aa and 66-114 aa).

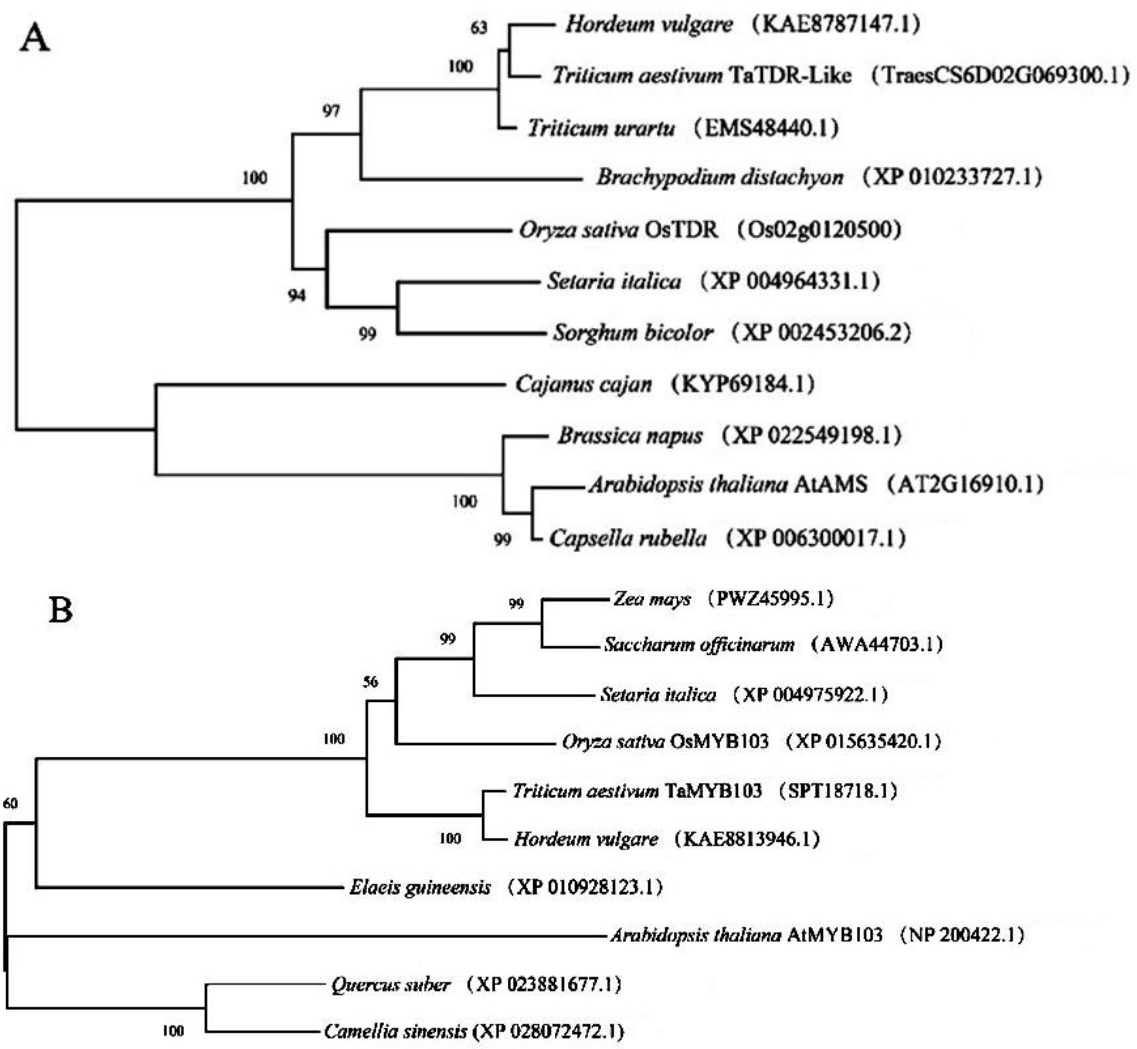

Figure S3. Phylogenetic tree based on the amino acid sequence of the protein TaTDRL and TaMYB103; (A) TaTDRL; (B) TaMYB103.

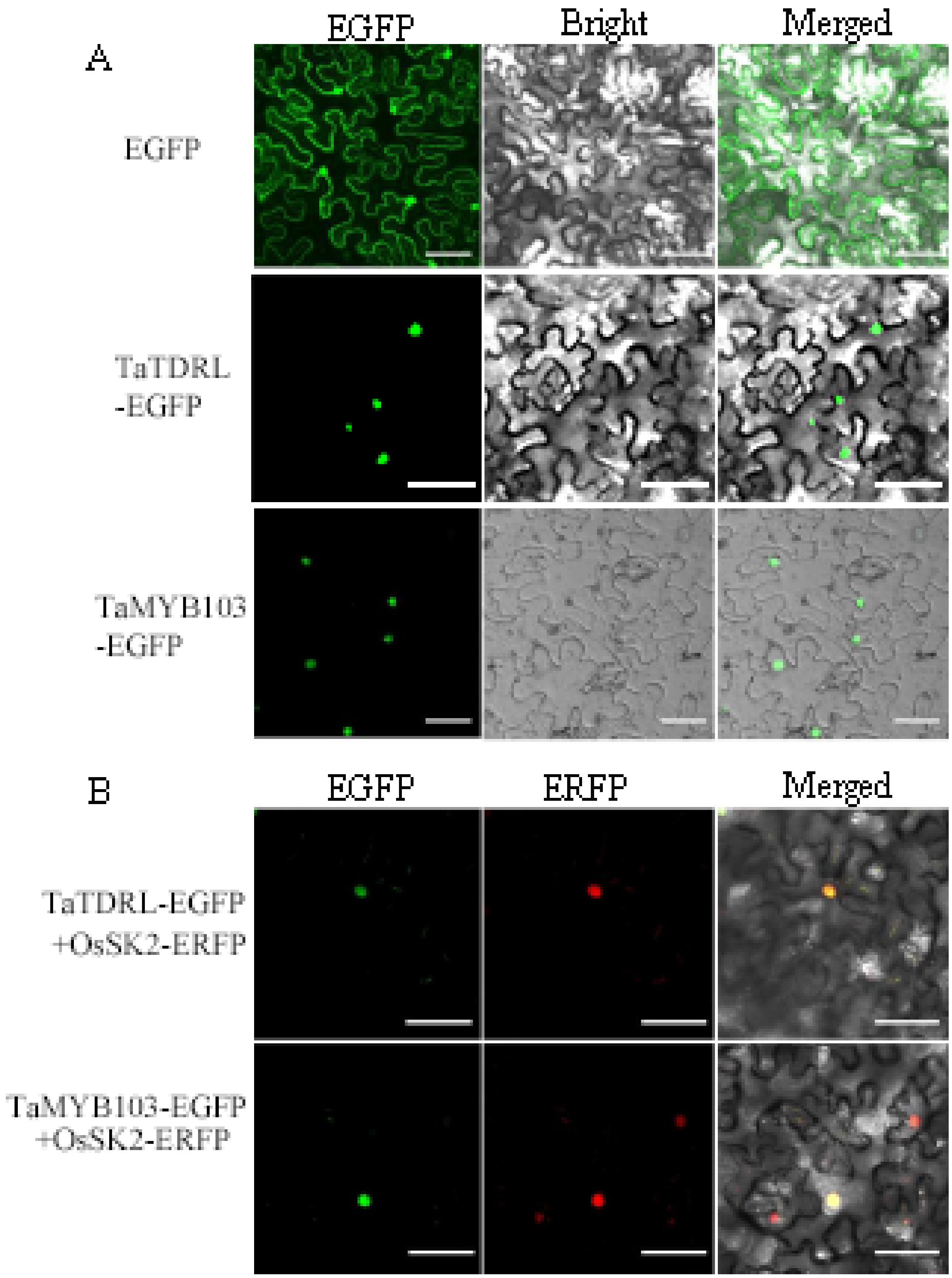

Figure S4. Subcellular localization of TaTDRL and TaMYB103 in *N. benthamiana* cells. (A) TaTDRL and TaMYB103 were fused with EGFP for subcellular localization analysis in tobacco leaves; (B) The reported nuclear marker gene *OsSK2* with ERFP (OsSK2-ERFP) and co-expressed it with TaTDRL-EGFP or TaMYB103-EGFP in tobacco leaves. The fluorescence (EGFP and ERFP), bright field, and merged images were obtained using a confocal microscope. Bar : 50μm.

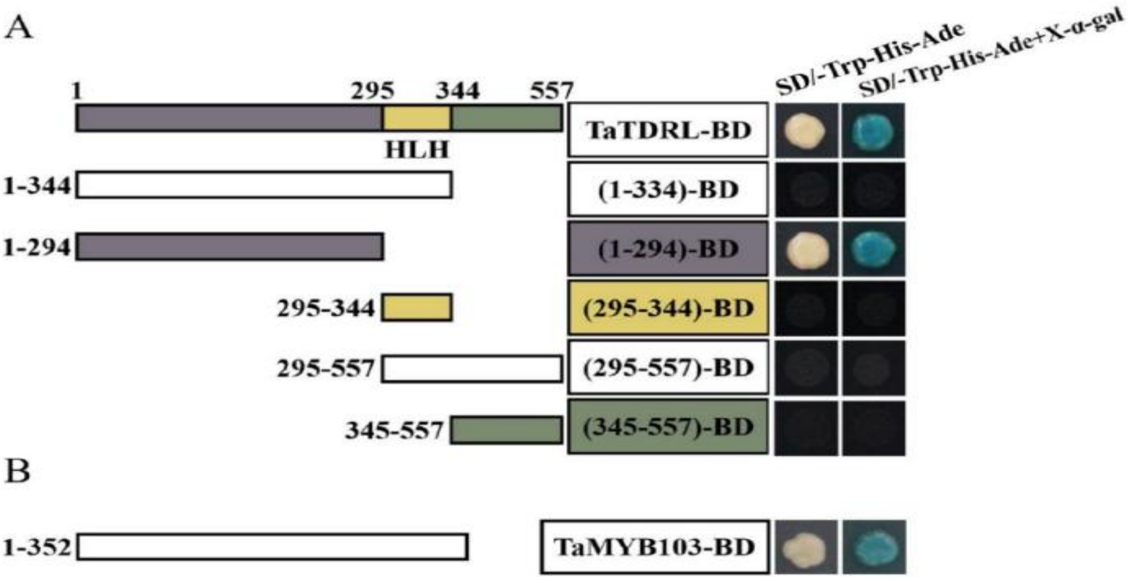

Figure S5. Yeast self-activating activity assay of TaTDRL and TaMYB103 protein; (A) Self-activating activity assay of the TaTDRL protein [45]; (B) Self-activating activity assay of the TaMYB103 protein. The transformed yeast cells were plated and grown on control plates (SD/-Trp) or selective plates (SD/-Trp-His-Ade + X-α -gal).

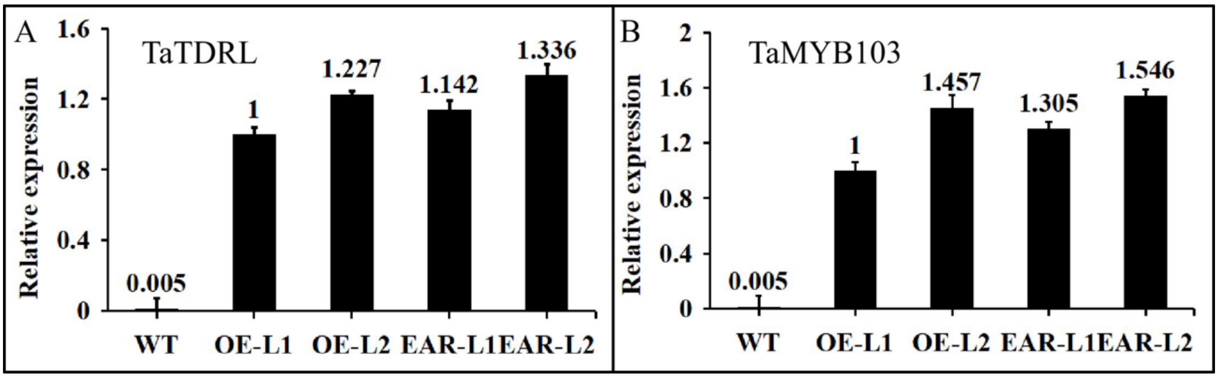

Figure S6. Identification of positive transgenic *Arabidopsis* plant; (A) qRT-PCR identifation of transgenic *Arabidopsis* plant with *TaTDRL*; (B) qRT-PCR identifation of transgenic *Arabidopsis* plant with *TaMYB103*.
